# Mild Cognitive Impairment or Attention-Deficit/Hyperactivity Disorder in Older Adults? A Cross Sectional Study

**DOI:** 10.3389/fpsyt.2021.737357

**Published:** 2021-09-20

**Authors:** Felippe Mendonca, Felipe Kenji Sudo, Gustavo Santiago-Bravo, Natalia Oliveira, Naima Assuncao, Fernanda Rodrigues, Rejane Soares, Victor Calil, Gabriel Bernardes, Pilar Erthal, Claudia Drummond, Fernanda Tovar-Moll, Paulo Mattos

**Affiliations:** ^1^D'Or Institute For Research and Education, Rio de Janeiro, Brazil; ^2^Department of Speech and Hearing Pathology, Federal University of Rio de Janeiro, Rio de Janeiro, Brazil; ^3^Department of Psychiatry and Forensic Medicine, Institute of Psychiatry, Federal University of Rio de Janeiro, Rio de Janeiro, Brazil

**Keywords:** attention deficit disorder with hyperactivity, cognitive dysfunction, dementia, neuropsychology, cognitive aging

## Abstract

**Background:** Attention-Deficit/Hyperactivity Disorder (ADHD) is a highly prevalent neurodevelopmental condition, which may be associated with life-enduring cognitive dysfunction. It has been hypothesized that age-related cognitive decline may overlap with preexisting deficits in older ADHD patients, leading to increased problems to manage everyday-life activities. This phenomenon may mimic neurodegenerative disorders, in particular Mild Cognitive Impairment (MCI). This cross-sectional study aims to assess cognitive and behavioral differences between older subjects with ADHD and MCI.

**Methods:** A total of 107 older participants (41 controls; 40 MCI and 26 ADHD; mean age = 67.60 ± 7.50 years; mean schooling = 15.14 ± 2.77 years; 65.4% females) underwent clinical, cognitive, and behavioral assessments by a multidisciplinary team at the Memory Clinic, D'Or Institute for Research and Education, Rio de Janeiro, Brazil. Mean scores in neuropsychological tasks and behavioral scales were compared across groups.

**Results:** Participants with ADHD showed poorer performances than controls in episodic memory and executive function with large effect-sizes. Performances were comparable between MCI and ADHD for all domains.

**Discussion:** MCI and ADHD in older individuals are dissociated clinical entities with overlapping cognitive profiles. Clinicians ought to be aware of these converging phenotypes to avoid misdiagnosis.

## Introduction

Originally regarded as exclusive to childhood and adolescence, Attention-Deficit/Hyperactivity Disorder (ADHD) has been recognized as a lifelong neurodevelopmental condition, persisting into old age in 3–4% of the cases (1, 2). However, available data on the natural history of the disorder beyond mid-adulthood are scarce, and the potential interactions between lifetime cognitive dysfunction, including attentional, executive and memory deficits, and age-related cognitive decline are still poorly understood (1, 3–6). Previous reports have suggested that late-life cognitive changes may overlap with these preexisting difficulties, leading to increased problems to manage everyday-life activities, which may mimic neurocognitive disorders, in particular Mild Cognitive Impairment (MCI) (5).

Alternatively, the idea that ADHD may accelerate or predispose to pathological neurogenerative processes is attractive, yet unproven (7). It could be argued that functional and clinical outcomes associated with the condition compared to unaffected individuals are too coincidental with established risk factors for dementia to be unrelated (8). Those include lower educational level (9) and increased prevalence of traumatic brain injury (10), obesity (11), smoking (12), alcohol-related disorders (13), depression (14), social isolation due to peer rejection (15) and physical inactivity (16). In addition, extensive data have indicated the contribution of oxidative stress and neuroinflammation to the pathophysiology of both ADHD and Alzheimer's Disease (17–20).

With that, characterizing the cognitive and behavioral profile of older subjects with ADHD is needed to allow further insight on potentially converging pathological mechanisms in neurodevelopmental and neurodegenerative disorders in late-life and to avoid misdiagnosis of conditions with resembling clinical manifestations. In addition, therapeutic implications of accurately distinguishing these clinical entities could exist, considering that the effectiveness of treatment for ADHD in adults has been proven (21). Hence, the present study aims to compare performances in standardized neuropsychological tests and severity of depression and anxiety across elderly individuals classified as normal controls, ADHD and MCI.

## Methods

### Sample

Participants were volunteers referred for clinical and cognitive assessment due to cognitive complaints by health care services and senior centers in Rio de Janeiro, Brazil. Subjects older than 55 years old and with four or more years of formal education were assessed between January 2014 and November 2020 in the Memory Clinic, D'Or Institute for Research and Education, Rio de Janeiro, Brazil. Those were eligible for the study if none of the following criteria were met: current delirium (22), history of severe psychiatric and neurological disorders (e.g., schizophrenia, bipolar disorder, intellectual disability, autism spectrum disorder, dementia or substance-related disorders) (22) and severe uncorrected sensorial deficits.

### Procedures

Participants underwent clinical, neuropsychological and language assessments by a multidisciplinary team. Initially, subjects were interviewed for current and past medical history by a physician. Neuropsychologists and speech-language therapists conducted neuropsychological and language protocols. Validated instruments for assessing cognitive changes in older Brazilian population were applied, including: (1) the Mini-Mental State Examination (MMSE), an estimate of global cognitive function (23, 24); (2) the Rey Auditory verbal-Learning Test (RAVLT), a measure of verbal episodic memory (25, 26); (3) the Block Design Test, which taps visuospatial abilities (27); (4) the Trail-making Test (TMT) parts A and B, which assesses visual tracking and cognitive flexibility (28); (5) the Digit Span Test, including the Forward and the Backwards subtests, which measures attention and working memory (29); (6) Semantic (animals) and phonemic (letters F, A, and S) verbal fluency test, which evaluates semantic memory and executive function (30). RAVLT subtests were analyzed, as follows: the sum of words retrieved during the immediate recall phase (RAVLT A1-A5); the number of correct items in the delayed recall task (RAVLT A7); the ratio A6/A5, corresponding to the retroactive interference index (RAVLT A6/A5); and the correctly recognized words during cued-recall task (RAVLT Rec).

Additionally, the Brazilian-Portuguese versions of the Geriatric Depression Scale (GDS) and the Geriatric Anxiety Scale (GAI) were applied to the participants (31–34). The presence of significant depressive symptoms was determined by the presence of 5 or more points in the GDS (32), whereas clinically relevant anxiety was defined as GAI > 13 (34). Functional status was considered preserved if participants scored 21 points in the adapted version for the Brazilian older population of the Lawton and Brody Scale (35).

### Classification

Diagnosis of ADHD was established by board-certified psychiatrists based on the DSM-5 criteria (22), independently from results on cognitive evaluation. The presence of DSM-5 inattentive and/or hyperactive/impulsive criteria, initiated during childhood, was determined through clinical interview. MCI was defined according to the DSM-5 criteria for Mild Neurocognitive Disorder, which comprises: (1) presence of cognitive complaint by the patient or relatives; (2) performances in neuropsychological tests below 1 SD from normative values for age and schooling and (3) functional status within normal range, as defined using the Lawton and Brody Scale (22, 35). Among MCI subjects, those presenting performances lower than 1 SD from normative data in one or more memory index were classified as Amnestic MCI.

### Statistical Analyses

Normality of data was assessed using Kolmogorov-Smirnov test. Mean differences in continuous sociodemographic variables were analyzed using one-way analysis of variance (ANOVA), followed by Games-Howell *post-hoc* test (36); or Kruskal-Wallis H test. Differences in distributions of categorical variables (sex, presence of significant depressive symptoms and anxiety) were assessed through Pearson's Chi-Square test (X^2^). Mean differences in neuropsychological performances across groups were assessed using analysis of covariance (ANCOVA—for normally distributed variables) or rank analysis of covariance (non-normally distributed variables) (37), adjusting for age, schooling, sex and severity of depressive symptoms (measured by the GDS), since groups differed in these measures. Effect-sizes were computed as partial eta-squared (partial η^2^); those values were interpreted according to benchmarks suggested by Cohen as follows: small, medium and large effect sizes were defined as partial η^2^ of 0.01, 0.06, and 0.14, respectively (38). Bonferroni correction was applied to avoid multiple testing issues when analyzing group-differences in cognitive tests. The alpha-level was divided by the number of comparisons; as a result, the level of significance was set at *p* < 0.004. All analyses were conducted using IBM Statistical Package for the Social Sciences (IBM SPSS v. 26).

### Ethics

All the participants provided an informed consent prior to the inclusion in the study. The project was approved by the Research Ethics Committee of the D'Or Institute under the protocol no. 226/11. The principles of the Resolution no. 510/2016 of the Brazil's National Health Council, which regulates research involving human beings in the country, as well as those of the latest version of the Declaration of Helsinki, were followed.

## Results

A total of 107 participants (mean age = 67.60 ± 7.50 years; mean schooling = 15.14 ± 2.77 years; 65.4% females) were included in the analyses. Sixty percent (*n* = 24) of participants in the MCI group were classified as amnestic MCI.

MCI group was significantly older and less educated than controls and ADHD participants. A higher proportion of females were found in the control group (82.9%) than among MCI (60.0%) and ADHD (46.2%) subjects (*p* < 0.05). Scores in GDS were significantly more elevated in ADHD participants than in controls (*p* < 0.05). Presence of clinically relevant depressive symptoms (GDS > 5) did not differ across groups (*p* = 0.14). Neither scores on GAI nor presence of clinical anxiety (GAI > 13) was distinct among diagnostics clusters. Table 1 depicts these results.

**Table 1:** Sociodemographic, behavioral, and cognitive characteristics of the sample.

	**Controls (** ***n*** **= 41)**	**MCI (** ***n*** **= 40)**	**ADHD (** ***n*** **= 26)**	**Indices**	***p*** **-value**
**Variables**	**Mean ± SD**	**Mean ± SD**	**Mean ± SD**		
Age (years)	65.60 ± 6.27	70.35 ± 6.63	66.53 ± 9.33	H = 10.881^a^, ^b^	<0.05
Schooling (years)	15.73 ± 2.30	14.00 ± 3.19	15.96 ± 2.19	H = 12.672^a^, ^b^	<0.05
Sex (%Females)	82.9	60.0	46.2	X^2^ = 10.340^a^, ^b^	<0.05
GDS	3.36 ± 3.29	4.27 ± 3.68	6.12 ± 4.68	H = 6.234^b^	<0.05
Depression (yes/no)	26.8	40.0	50.0	X^2^ = 3.836	0.14
GAI	5.95 ± 4.94	6.90 ± 5.75	9.08 ± 5.20	H = 5.200	0.07
Anxiety (yes/no)	14.60	17.50	26.90	X^2^ = 1.641	0.44

a *Controls ≠ MCI*.

b *Controls ≠ ADHD*.

*MCI, Mild Cognitive Impairment; ADHD, Attention-Deficit/Hyperactivity Disorder; GDS, Geriatric Depression Scale; GAI, Geriatric Anxiety Scale*.

After adjustment for age, sex, years of schooling and GDS, mean scores in all memory indices (RAVLT A1-A5, RAVLT A7, RAVLT A6/A5, and RAVLT Rec), TMT B, Digit Span Forward and Digit Span Backwards were significantly different between controls and MCI subjects. Controls performed significantly better than ADHD group in RAVLT A1-A5, RAVLT A7, RAVLT Rec, TMT B and Digit Span Forward. Except for Digit Span Backwards (partial η^2^ = 0.13), effect-sizes were large for all these variables (partial η^2^ > 0.14). No mean difference was observed between ADHD and MCI in the cognitive tasks. Those findings are shown in Table 2.

**Table 2:** Neuropsychological performances across groups with adjustments for age, sex, schooling, and depressive symptoms.

	**Controls (** ***n*** **= 41)**	**MCI (** ***n*** **= 40)**	**ADHD (** ***n*** **= 26)**	**Index**	***p*** **-value**	**Effect-size**
**Tasks**	**Mean ± SD**	**Mean ± SD**	**Mean ± SD**			
MMSE	28.46 ± 1.30	26.77 ± 1.90	27.65 ± 1.85	F = 2,897	0.06	Partial η^2^ = 0.05
Block Design	31.56 ± 8.54	23.92 ± 9.30	27.20 ± 12.85	F = 3,146	0.06	Partial η^2^ = 0.05
RAVLT A1-A5	51.21 ± 6.98	36.52 ± 9.27	42.19 ± 10.40	F = 1,6045^a^, ^b^	<0.001^*^	Partial η^2^ = 0.24
RAVLT A7	10.90 ± 2.37	5.87 ± 3.54	7.96 ± 3.41	F = 1,4961^a^, ^b^	<0.001^*^	Partial η^2^ = 0.23
RAVLT A6/A5	0.84 ± 0.14	0.60 ± 0.27	0.73 ± 0.22	F = 4,440^a^	0.01	Partial η^2^ = 0.08
RAVLT Rec	14.95 ± 2.41	11.85 ± 3.73	9.00 ± 6.35	F = 11,393^a^, ^b^	<0.001^*^	Partial η^2^ = 0.18
TMT A	38.31 ± 12.01	58.05 ± 26.65	47.75 ± 21.86	F = 3,982^a^	0.02	Partial η^2^ = 0.07
TMT B	89.36 ± 30.57	154.16 ± 67.87	133.34 ± 72.06	F = 9,863^a^, ^b^	<0.001^*^	Partial η^2^ = 0.16
Digit Span Forward	8.80 ± 2.08	7.27 ± 1.93	6.61 ± 2.07	F = 9,211^a^, ^b^	<0.001^*^	Partial η^2^ = 0.15
Digit Span Backwards	6.29 ± 1.81	4.60 ± 1.69	5.11 ± 1.75	F = 7,885^a^	0.001^*^	Partial η^2^ = 0.13
Semantic VF	18.70 ± 4.81	16.28 ± 4.78	15.36 ± 5.25	F = 3,222^b^	0.04	Partial η^2^ = 0.06
Phonemic VF	42.73 ± 18.73	36.69 ± 15.25	33.16 ± 13.57	F = 2,082	0.13	Partial η^2^ = 0.04

a *Controls ≠ MCI*.

b *Controls ≠ ADHD*.

* *Significant at the 0.004 level*.

*MCI, Mild Cognitive Impairment; ADHD, Attention-Deficit/Hyperactivity Disorder; MMSE, Mini-Mental State Examination; RAVLT, Rey Auditory verbal-Learning Test; TMT A, Trail-making test part A; TMT B, Trail-Making Test part B; VF, Verbal fluency*.

## Discussion

In this study, a pattern of amnestic-dysexecutive neuropsychological deficits was evidenced in older subjects with ADHD in comparison with normal controls, after controlling for age, years of schooling, sex and severity of depressive symptoms. However, task performances in this group were not distinguishable from MCI, suggesting that these conditions manifest as overlapping cognitive syndromes.

Verbal memory impairments in ADHD were identified for acquisition, delayed recall and recognition processes in relation to controls, which is in line with previous systematic and scoping reviews examining cognitive deficits in adults with the disorder (39, 40). It has been presumed that poor memory strategy selection in those individuals due to a disrupted phonological loop may lead to inefficient encoding of the material to be learned. With the deficient conversion of verbal stimuli into memory, performances in tasks involving retrieval of information both spontaneously and in response to verbal cues predictably fall below the average levels (39, 41).

In addition, scores in executive function tasks were comparable between ADHD and MCI groups. Accordingly, previous reports suggested that severity of frontal-executive impairments in ADHD, whether inherent of the disorder or exacerbated by age-related cognitive decline, might be compatible with subtle, but measurable difficulties to perform everyday life activities, which might mimic MCI presentation (41, 42). Longitudinal investigation of executive control in this diagnostic group may clarify about the contribution of aging to cognitive load and disability in ADHD.

Several limitations of the present study ought to be discussed. Given that awareness of ADHD, especially of later-life cases, has only been recently widespread (43), none of the participants with the condition had been given a diagnosis before the initial assessment in the study. Detection of the disorder was based on retrospective reports of DSM-5 childhood-onset of inattentive and/or hyperactive/impulsive criteria. Evidence of stability of ADHD traits through lifespan might suggest the validity of classification measures developed for younger populations in older samples (44), but more studies addressing this issue are needed (2). Furthermore, the etiological heterogeneity of MCI identified using solely clinical parameters has been widely recognized (45). Future studies applying biomarkers of neurodegenerative and cerebrovascular diseases might reduce selection bias. Finally, the small sample size, the tertiary setting, and the cross-sectional design might also affect validity of our results. Hence, large longitudinal community-based research is needed to confirm our findings.

In conclusion, our data suggest that MCI and ADHD are dissociated clinical entities with overlapping cognitive profiles. Considering the distinct therapeutic implications of the detection of these conditions, clinicians ought to be aware of these converging phenotypes to avoid misdiagnosis. A high level of suspicious and detailed history taking is crucial to allow the differential diagnosis of these disorders.

## Data Availability Statement

The datasets presented in this study can be found in online repositories. The names of the repository/repositories and accession number(s) can be found below: https://doi.org/10.6084/m9.figshare.14916888.

## Ethics Statement

The studies involving human participants were reviewed and approved by Research Ethics Committee of the IDOR. The patients/participants provided their written informed consent to participate in this study.

## Author Contributions

All authors listed have made a substantial, direct and intellectual contribution to the work, and approved it for publication.

## Funding

This research was supported by intramural grants from the D'Or Institute for Research and Education and Rede D'Or São Luiz Hospital Network, Conselho Nacional de Pesquisa, Coordenação de Aperfeiçoamento de Pessoal de Ensino Superior and Fundação de Amparo à Pesquisa do Rio de Janeiro.

## Conflict of Interest

The authors declare that the research was conducted in the absence of any commercial or financial relationships that could be construed as a potential conflict of interest.

## Publisher's Note

All claims expressed in this article are solely those of the authors and do not necessarily represent those of their affiliated organizations, or those of the publisher, the editors and the reviewers. Any product that may be evaluated in this article, or claim that may be made by its manufacturer, is not guaranteed or endorsed by the publisher.

## References

[1] Kooij JJS Michielsen M Kruithof H Bijlenga D. ADHD in old age: a review of the literature and proposal for assessment and treatment. Expert Rev Neurotherap. (2016) 16:1371–81. 10.1080/14737175.2016.1204914 27334252

[2] Surman CBH Goodman DW. Is ADHD a valid diagnosis in older adults? ADHD Atten Def Hyp Disord. (2017) 9:161–8. 10.1007/s12402-017-0217-x 28124223

[3] Schoechlin C Engel R. Neuropsychological performance in adult attention-deficit hyperactivity disorder: meta-analysis of empirical data. Arch Clin Neuropsychol. (2005) 20:727–44. 10.1016/j.acn.2005.04.005 15953706

[4] Patros CHG Tarle SJ Alderson RM Lea SE Arrington EF. Planning deficits in children with attention-deficit/hyperactivity disorder (ADHD): a meta-analytic review of tower task performance. Neuropsychology. (2019) 33:425–44. 10.1037/neu0000531 30688493

[5] Callahan BL Bierstone D Stuss DT Black SE. Adult ADHD: risk factor for dementia or phenotypic mimic? Front Aging Neurosci. (2017) 9:260. 10.3389/fnagi.2017.00260 28824421 PMC5540971

[6] Faraone SV Banaschewski T Coghill D Zheng Y Biederman J Bellgrove MA. The world federation of ADHD international consensus statement: 208 evidence-based conclusions about the disorder. Neurosci Biobehav Rev. (2021) 128:789–818. 10.1016/j.neubiorev.2021.01.022 33549739 PMC8328933

[7] Ivanchak N Abner EL Carr SA Freeman SJ Seybert A Ranseen J. Attention-Deficit/hyperactivity disorder in childhood is associated with cognitive test profiles in the geriatric population but not with mild cognitive impairment or Alzheimer's disease. J Aging Res. (2011) 2011:1–7. 10.4061/2011/729801 21822493 PMC3142705

[8] Livingston G Huntley J Sommerlad A Ames D Ballard C Banerjee S. Dementia prevention, intervention, and care: 2020 report of the Lancet Commission. Lancet. (2020) 396:413–46. 10.1016/S0140-6736(20)30367-6 32738937 PMC7392084

[9] Fredriksen M Dahl AA Martinsen EW Klungsoyr O Faraone SV Peleikis DE. Childhood and persistent ADHD symptoms associated with educational failure and long-term occupational disability in adult ADHD. ADHD Atten Def Hyp Disord. (2014) 6:87–99. 10.1007/s12402-014-0126-1 24497125 PMC4033786

[10] Adeyemo BO Biederman J Zafonte R Kagan E Spencer TJ Uchida M. Mild traumatic brain injury and ADHD: a systematic review of the literature and meta-analysis. J Atten Disord. (2014) 18:576–84. 10.1177/1087054714543371 25047040

[11] Cortese S Moreira-Maia CR St. Fleur D Morcillo-Peñalver C Rohde LA Faraone SV. Association between ADHD and obesity: a systematic review and meta-analysis. AJP. (2016) 173:34–43. 10.1176/appi.ajp.2015.15020266 26315982

[12] van Amsterdam J van der Velde B Schulte M van den Brink W. Causal factors of increased smoking in ADHD: a systematic review. Substance Use Misuse. (2018) 53:432–45. 10.1080/10826084.2017.1334066 29039714

[13] Luderer M Sick C Kaplan-Wickel N Reinhard I Richter A Kiefer F. Prevalence estimates of ADHD in a sample of inpatients with alcohol dependence. J Atten Disord. (2020) 24:2072–83. 10.1177/1087054717750272 29308693

[14] Sandstrom A Perroud N Alda M Uher R Pavlova B. Prevalence of attention-deficit/hyperactivity disorder in people with mood disorders: a systematic review and meta-analysis. Acta Psychiatr Scand. (2021) 143:380–91. 10.1111/acps.13283 33528847

[15] Mrug S Molina BSG Hoza B Gerdes AC Hinshaw SP Hechtman L. Peer rejection and friendships in children with attention-deficit/hyperactivity disorder: contributions to long-term outcomes. J Abnorm Child Psychol. (2012) 40:1013–26. 10.1007/s10802-012-9610-2 22331455 PMC3384771

[16] Mercurio LY Amanullah S Gill N Gjelsvik A. Children with ADHD engage in less physical activity. J Atten Disord. (2021) 25:1187–95. 10.1177/1087054719887789 31838947

[17] Joseph N Zhang-James Y Perl A Faraone SV. Oxidative stress and ADHD: a meta-analysis. J Atten Disord. (2015) 19:915–24. 10.1177/1087054713510354 24232168 PMC5293138

[18] Leffa DT Torres ILS Rohde LA. A review on the role of inflammation in attention-deficit/hyperactivity disorder. Neuroimmunomodulation. (2018) 25:328–33. 10.1159/000489635 29874674

[19] Butterfield DA Halliwell B. Oxidative stress, dysfunctional glucose metabolism and Alzheimer disease. Nat Rev Neurosci. (2019) 20:148–60. 10.1038/s41583-019-0132-6 30737462 PMC9382875

[20] Leng F Edison P. Neuroinflammation and microglial activation in Alzheimer disease: where do we go from here? Nat Rev Neurol. (2021) 17:157–72. 10.1038/s41582-020-00435-y 33318676

[21] De Crescenzo F Cortese S Adamo N Janiri L. Pharmacological and non-pharmacological treatment of adults with ADHD: a meta-review. Evid Based Mental Health. (2017) 20:4–11. 10.1136/eb-2016-102415 27993933 PMC10699262

[22] American Psychiatric Association. Diagnostic and Statistical Manual of Mental Disorders. 5th ed. Arlington, VA: American Psychiatric Association (2013). Available online at: 10.1176/appi.books.9780890425596.dsm05 (accessed February 18, 2021).

[23] Folstein MF Folstein SE McHugh PR. Mini-mental state. J Psychiatr Res. (1975) 12:189–98. 10.1016/0022-3956(75)90026-6 1202204

[24] Brucki SMD Nitrini R Caramelli P Bertolucci PHF Okamoto IH. Sugestões para o uso do mini-exame do estado mental no Brasil. Arq Neuro Psiquiatr. (2003) 61:777–81. 10.1590/S0004-282X2003000500014 14595482

[25] Rey A. L'Examen Clinique en Psychologie. Paris: Press Universitaire de France (1958).

[26] Paula JJ de Melo LPC Nicolato R Moraes EN de Bicalho MA Hamdan AC. Fidedignidade e validade de construto do Teste de Aprendizagem Auditivo-Verbal de Rey em idosos brasileiros. Rev Psiquiatr Clín. (2012) 39:19–23. 10.1590/S0101-60832012000100004

[27] Wechsler D. Examiner's manual: Wechsler Adult Intelligence Scale-Revised. New York, NY: Psychological Corporation (1981).

[28] Army Individual Test Battery. Manual of Directions and Scoring. Washington, DC: War Department, Adjunt General's Office Trail (1944).

[29] Wechsler D. WAIS-III: Administration and Scoring Manual. San Antonio: Psychological Corporation (1997).

[30] Martyr A Clare L Nelis SM Marková IS Roth I Woods RT. Verbal fluency and awareness of functional deficits in early-stage dementia. Clin Neuropsychol. (2012) 26:501–19. 10.1080/13854046.2012.665482 22394254

[31] Yesavage JA Brink TL Rose TL Lum O Huang V Adey M. Development and validation of a geriatric depression screening scale: a preliminary report. J Psychiatr Res. (1982) 17:37–49. 10.1016/0022-3956(82)90033-4 7183759

[32] Paradela EMP Lourenço RA Veras RP. Validação da escala de depressão geriátrica em um ambulatório geral. Rev Saúde Pública. (2005) 39:918–23. 10.1590/S0034-89102005000600008 16341401

[33] Pachana NA Byrne GJ Siddle H Koloski N Harley E Arnold E. Development and validation of the geriatric anxiety inventory. IPG. (2007) 19:103. 10.1017/S1041610206003504 16805925

[34] Massena PN de Araújo NB Pachana N Laks J de Pádua AC. Validation of the Brazilian portuguese version of geriatric anxiety inventory – GAI-BR. Int Psychogeriatr. (2015) 27:1113–119. 10.1017/S1041610214001021 24946782

[35] dos Santos R Virtuoso-Junior J. Confiabilidade da versão brasileira da escala de atividades instrumentais da vida diária. Revista Brasileira em Promoção da Saúde. (2008) 21:290–6. 10.5020/18061230.2008.p290

[36] Sauder DC DeMars CE. An updated recommendation for multiple comparisons. Adv Methods Pract Psychol Sci. (2019) 2:26–44. 10.1177/2515245918808784

[37] Quade D. Rank analysis of covariance. J Am Statist Assoc. (1967) 62:1187–200. 10.1080/01621459.1967.10500925

[38] Cohen J. Statistical Power Analysis for the Behavioral Sciences. 2nd ed. New York, NY: LAWRENCE ERLBAUM ASSOCIATES (1988). Available online at: http://www.utstat.toronto.edu/~brunner/oldclass/378f16/readings/CohenPower.pdf (accessed August 13, 2021).

[39] Skodzik T Holling H Pedersen A. Long-term memory performance in adult ADHD: a meta-analysis. J Atten Disord. (2017) 21:267–83. 10.1177/1087054713510561 24232170

[40] Hervey AS Epstein JN Curry JF. Neuropsychology of adults with attention-deficit/hyperactivity disorder: a meta-analytic review. Neuropsychology. (2004) 18:485–503. 10.1037/0894-4105.18.3.485 15291727

[41] Callahan BL Ramakrishnan N Shammi P Bierstone D Taylor R Ozzoude M. Distinct cognitive and neuroimaging profiles in later-life attention deficit/hyperactivity disorder and mild cognitive impairment. Res. Square [Preprint]. (2020). 10.21203/rs.3.rs-91495/v1 PMC9066671 34784815

[42] Marshall GA Rentz DM Frey MT Locascio JJ Johnson KA Sperling RA Alzheimer's Disease Neuroimaging Initiative. Executive function and instrumental activities of daily living in mild cognitive impairment and Alzheimer's disease. Alzheimers Dementia. (2011) 7:300–8. 10.1016/j.jalz.2010.04.005 21575871 PMC3096844

[43] Sharma MJ Lavoie S Callahan BL. A call for research on the validity of the age-of-onset criterion application in older adults being evaluated for ADHD: a review of the literature in clinical and cognitive psychology. Am J Geriatr Psychiatry. (2021) 29:669–78. 10.1016/j.jagp.2020.10.016 33191098

[44] Semeijn EJ Comijs HC de Vet HCW Kooij JJS Michielsen M Beekman ATF. Lifetime stability of ADHD symptoms in older adults. Atten Def Hyp Disord. (2016) 8:13–20. 10.1007/s12402-015-0178-x 26068984

[45] Jack CR Bennett DA Blennow K Carrillo MC Dunn B Haeberlein SB. NIA-AA research framework: toward a biological definition of Alzheimer's disease. Alzheimers Dementia. (2018) 14:535–62. 10.1016/j.jalz.2018.02.018 29653606 PMC5958625

